# First Report of *bla*_CTX*‐*M‐15_ in a Marine Thermotolerant *Enterobacter* Isolate From Bangladesh: Genomic Insights Into Halotolerance and Antimicrobial Resistance

**DOI:** 10.1002/mbo3.70425

**Published:** 2026-09-29

**Authors:** Md. Arifur Rahman, Rafid Muntasir Ul Azam, Md. Sazib Hossain, Samina Momtaz, Anowara Begum, Munawar Sultana

**Affiliations:** ^1^ Department of Microbiology University of Dhaka Dhaka Bangladesh; ^2^ Department of Microbiology, School of Life Sciences BRAC University Dhaka Bangladesh; ^3^ Department of Microbiology Jahangirnagar University Dhaka Bangladesh

**Keywords:** antimicrobial resistance, Bangladesh, blaCTX‐M‐15, Enterobacter kobei, ESBL, halotolerance, marine microbiome, mobile genetic elements, One Health

## Abstract

Marine ecosystems are becoming important reservoirs for antimicrobial resistance (AMR). However, genomic data on ESBL (Extended‐Spectrum Beta‐Lactamase)‐producing *Enterobacter* species from marine environments are limited. The widespread *bla*_CTX*‐*M‐15_ gene, usually found in clinical and wastewater settings, has not been reported in marine *Enterobacter* from Bangladesh. A thermotolerant, lactose‐fermenting isolate (LB01) from the Bay of Bengal seawater near Laboni Beach, Cox's Bazar, was isolated and identified as *Enterobacter kobei* based on > 99.4% average nucleotide identity and phylogenetic clustering. Its 4.62 Mb genome (55.05% GC) encoded 4410 genes, including *bla*_CTX*‐*M‐15_, *bla*_ACT*‐*9_, and *qnrS1*, with *bla*_CTX*‐*M‐15_ located next to an ISEc9‐like insertion sequence, indicating a mobilizable resistance locus. The genome also contained efflux systems (*acrAB, mdtABC, oqxAB*), adhesion and iron uptake genes (*fim, flh, ent, fep, chu*), and comprehensive halotolerance modules (*proVWX, betT, nhaA, kdpABC, mscL/S*) consistent with its growth in up to 7% NaCl. This is the first genomic characterization of a marine *E. kobei* carrying *bla*_CTX*‐*M‐15_ resistant determinant in Bangladesh, showing convergence of resistance, virulence, and halotolerance traits that support persistence in saline, human‐impacted environments. The findings broaden the ecological scope of clinically relevant ESBL genes and highlight the importance of coastal ecosystems as overlooked nodes in the global AMR network within a One Health framework.

## Graphical abstract

Marine‐derived *Enterobacter kobei*, exhibiting thermotolerance and salt tolerance, is ESBL‐producing and harbors AMR genes (*bla*_*CTX‐M‐15*_, *qnrS1*) and mobile genetic elements, indicating environmental spread of antimicrobial resistance.

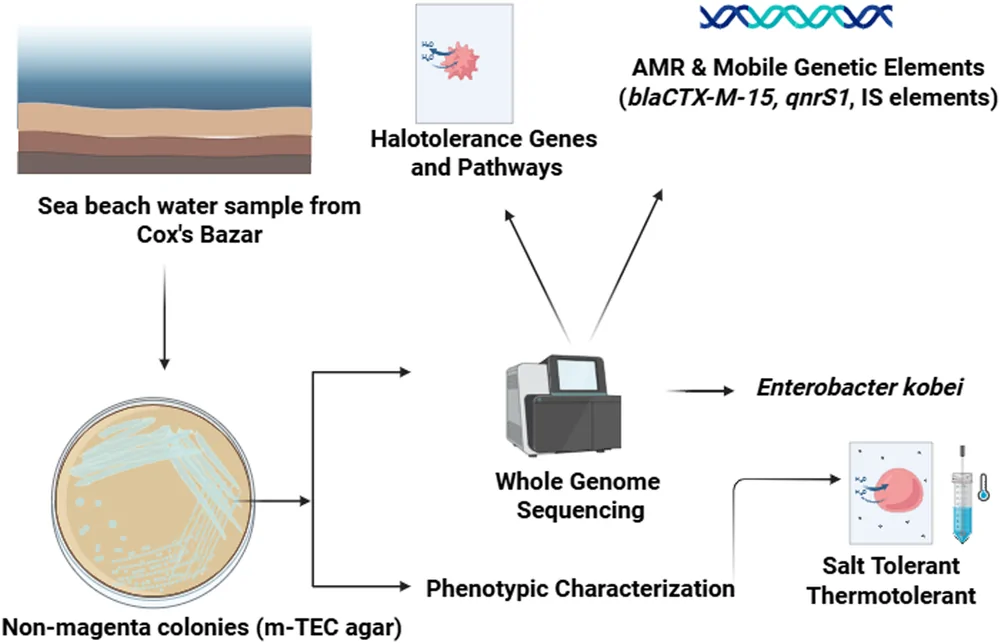

## 1 Introduction

Antimicrobial resistance (AMR) is a significant global health issue and a concern in the One Health approach connecting human, animal, and environmental sectors (Brown et al. 2024; Kraemer et al. 2019). Environmental waters, such as rivers, estuaries, and marine systems, serve as reservoirs and pathways for antibiotic‐resistant bacteria (ARB) and antibiotic resistance genes (ARGs), thereby promoting the persistence, selection, and horizontal transfer of resistance factors across different ecological areas (Amarasiri et al. 2020; Meradji et al. 2025; Singh et al. 2022). The environmental spread of antibiotic‐resistant genes (ARGs) results from multiple sources, including sewage, agricultural runoff, aquaculture, and hospital waste and is now seen as a crucial part of the global AMR crisis (Larsson and Flach 2022).

Among β‐lactamases, CTX‐M‐type extended‐spectrum β‐lactamases (ESBLs) have become the most prevalent and clinically significant family, displacing earlier TEM and SHV variants in many regions (Castanheira et al. 2021). Within this group, *bla*_CTX*‐*M‐15_ (a member of the CTX‐M‐1 cluster) has emerged as globally dominant among diverse *Enterobacterales* in both healthcare and community settings. Its widespread dissemination is largely driven by association with epidemic clones (e.g., *Escherichia coli* ST131, *Klebsiella pneumoniae* ST15) and by its frequent linkage with mobile genetic elements (MGEs) such as ISEcp1 and IS26, which promote horizontal gene transfer and integration into plasmid or chromosomal backbones (Husna et al. 2023; Yu et al. 2024).

The genus *Enterobacter*, within the order Enterobacterales, comprises ubiquitous opportunistic pathogens responsible for a range of nosocomial and community infections (Ramirez & Giron, 2023). Clinically relevant *Enterobacter* species frequently carry multiple resistance determinants and can persist in environmental reservoirs; their presence in aquatic and food‐associated environments therefore represents a plausible route for ARG transmission to humans (Davin‐Regli et al. 2019).

In Bangladesh, which has dense coastal populations, extensive aquaculture, and often inadequate wastewater treatment infrastructure, there is increasing evidence of environmental contamination by ESBL producers. Surveillance studies have documented high rates of ESBL‐producing *Enterobacterales* in clinical isolates, and detection of ESBL genes (including CTX‐M types) in surface waters and seafood (Firdous et al. 2025). Environmental water studies in Dhaka have isolated *Enterobacter cloacae* and others producing CTX‐M and SHV ESBLs from urban surface water and rivers, showing that water bodies are acting as reservoirs of clinically relevant ESBL genes (Haque et al. 2014).

Despite increasing reports of ESBL‐producing *Enterobacterales* in aquatic environments, the occurrence and genomic context of *bla*_CTX*‐*M‐15_ in marine *Enterobacter* species remain poorly understood, particularly in Bangladesh. We hypothesized that human‐impacted coastal environments may harbor clinically relevant resistance determinants in environmental *Enterobacter* populations.

To test this, we performed whole‐genome sequencing and functional characterization of a marine‐derived Enterobacter isolate to (i) determine its taxonomic identity, (ii) investigate the genetic context and mobility potential of *bla*_CTX*‐*M‐15_, (iii) characterize its resistome and virulence‐associated traits, and (iv) assess genomic features supporting halotolerance and environmental persistence. This research broadens current understanding of the ecological spread of *bla*_CTX*‐*M‐15_ and highlights the importance of marine ecosystems as underexplored reservoirs of clinically relevant AMR within a One Health perspective.

## 2 Materials and Methods

### 2.1 Sample Collection and Bacterial Isolation

Seawater samples were collected from the coastal site of Laboni Beach (21.4274925° N, 91.9708149° E) along the Bay of Bengal, Bangladesh, in February 2024. Physicochemical characteristics of the sampling site were determined at the time of collection, including salinity, pH, chemical oxygen demand (COD), major ions, nutrients, and trace metals. The measured values were as follows: salinity 30 PSU, pH 7.2, COD 910 mg/L, chloride 19,500 mg/L, sulphate 4300 mg/L, total phosphorus 0.2 mg/L, and TKN 0 mg/L. Heavy metal concentrations included Pb (0.216 ppm), Cd (0.03 ppm), Cr (0.005 ppm), Cu (0.02 ppm), Ni (0.119 ppm), and Zn (0.13 ppm), with Hg below detectable limits. Samples were transported to the laboratory in sterile containers on ice and processed within 6 h of collection. For bacterial isolation, seawater aliquots were serially diluted and plated onto m‐TEC ChromoSelect Agar (NutriSelect Plus; Sigma‐Aldrich/Merck Millipore), a US EPA‐recommended selective and differential medium formulated for the detection of thermotolerant *Escherichia coli*. The medium contains selective agents such as bile salts and chromogenic substrates that enable differentiation based on β‐glucuronidase activity. Plates were incubated at 44.5°C for 22–24 h. While this medium is optimized for *E. coli*, other thermotolerant members of the order *Enterobacterales*, including *Enterobacter* spp., may grow under these conditions due to shared physiological traits. Colonies displaying morphology distinct from the characteristic red‐magenta appearance of *Escherichia coli* on m‐TEC agar, including non‐magenta colonies, were selected for further investigation. These isolates were subsequently subcultured on MacConkey agar, where they produced pink, mucoid, circular colonies consistent with lactose fermentation. A single colony was purified on nutrient agar and subjected to standard biochemical tests, including indole production, citrate utilization, and Kligler iron agarKligler iron agar (KIA) reactions, to establish its presumptive identity, following established protocols described in Bergey's Manual of Systematic Bacteriology. Final confirmation of the isolate was achieved by 16S rRNA gene sequencing (Lane 1991).

### 2.2 Antimicrobial Susceptibility Testing

The antimicrobial susceptibility profile was determined by the Kirby–Bauer disk diffusion method on Mueller–Hinton agar following CLSI guidelines (CLSI, 2025). Discs tested included ampicillin (10 μg), cefotaxime (30 μg), ceftazidime (30 μg), ceftriaxone (30 μg), imipenem (10 μg), ciprofloxacin (5 μg), gentamicin (10 μg), tetracycline (30 μg), nalidixic acid (30 μg), and trimethoprim–sulfamethoxazole (25 μg). The inhibition zone diameters were measured and interpreted according to Clinical and Laboratory Standards Institute (CLSI) guidelines. The detailed antimicrobial susceptibility profile of isolate LB01, along with the corresponding CLSI breakpoint criteria used for interpretation, is presented in Table 1. *Escherichia coli* ATCC 25922 was used as the quality control strain to ensure the accuracy and reliability of the assay.

**Table 1:** Antimicrobial susceptibility profile of the isolate LB01.

Class	Antibiotic (disk content)	Zone diameter (mm)	CLSI breakpoint (mm) S/I/R	Interpretation
Penicillins	Ampicillin (10 µg)	0	≥ 17/14–16/ ≤ 13	R
Cephalosporins (3rd gen)	Cefotaxime (30 µg)	9	≥ 26/23–25/ ≤ 22	R
	Ceftazidime (30 µg)	0	≥ 21/18–20/ ≤ 17	R
	Ceftriaxone (30 µg)	9	≥ 23/20–22/ ≤ 19	R
Fluoroquinolones	Ciprofloxacin (5 µg)	26	≥ 26/22–25/ ≤ 21	S
Quinolones (1st gen)	Nalidixic acid (30 µg)	20	≥ 19/14–18/ ≤ 13	S
Aminoglycosides	Gentamicin (10 µg)	20	≥ 18/15–17/ ≤ 14	S
Carbapenems	Imipenem (10 µg)	25	≥ 23/20–22/ ≤ 19	S
Tetracyclines	Tetracycline (30 µg)	17	≥ 15/12–14/ ≤ 11	S
Sulfonamides/Folate inhibitors	Trimethoprim–sulfamethoxazole (SXT/TR)	18	≥ 16/11–15/ ≤ 10	S

### 2.3 Salt Tolerance and Phenotypic Assays

Salt tolerance was assessed across a NaCl gradient of 0, 2, 5, 7, and 10% (w/v). These concentrations encompass the environmentally relevant salinity of the sampling site (~ 30 PSU, equivalent to approximately 3.0% total dissolved salts, primarily NaCl) and extend to higher levels to determine the upper tolerance limit. This range is consistent with NaCl gradients (2%–10%) commonly used to characterize halotolerance in *Enterobacter* spp. (Tian et al. 2025).

The strain LB01 was inoculated into Nutrient Broth (NB; HiMedia, India) supplemented with NaCl at concentrations of 2%, 5%, 7%, and 10% (w/v) and incubated at 37°C for 24 h under shaking conditions (150 rpm). Each flask contained 10 mL medium inoculated with 1% (v/v) of an overnight culture (OD_600_ ≈ 1.0). Growth was assessed both visually and spectrophotometrically by measuring optical density at 600 nm (Thermo Scientific, USA). To confirm salt tolerance, cultures showing turbidity were streaked onto Nutrient Agar (NA) plates containing corresponding NaCl concentrations and incubated at 37°C for 24 h to observe colony formation. Hemolytic activity was tested on 5% sheep blood agar (HiMedia, India) by streaking the isolate and incubating at 37°C for 24–48 h. *E. coli* DH5α (ATCC 53868) was used as a non‐halotolerant laboratory control strain.

### 2.4 Genomic DNA Extraction and Whole‐Genome Sequencing

Genomic DNA was extracted from overnight cultures grown in Nutrient Broth using the TIANamp Genomic DNA Kit (Tiangen Biotech, China) following the manufacturer's instructions for cells and microbial cultures. DNA quality and concentration were assessed using a NanoDrop spectrophotometer (Thermo Fisher Scientific, USA). Sequencing libraries were prepared using the Nextera XT DNA Library Preparation Kit (Illumina, USA) and sequenced on an Illumina platform to generate 2 × 150 bp paired‐end reads.

### 2.5 Quality Control, Genome Assembly, and Annotation

Raw reads were assessed using FastQC (Andrews 2010) and trimmed with Trimmomatic v0.39 (Bolger et al. 2014) with the following parameters: ILLUMINACLIP:TruSeq. 3‐PE. fa:2:30:10, LEADING:20, TRAILING:20, SLIDINGWINDOW:4:20, and MINLEN:50. De novo assembly was performed using SPAdes v3.15.5 (Bankevich et al. 2012) with default parameters and multiple k‐mer sizes (21, 33, 55, 77), and the “‐‐careful” option enabled to reduce mismatches. Genome annotation was carried out with the RAST toolkit (RASTtk) (Brettin et al. 2015) under default settings, and further annotated with Prokka v1.14.6 (Seemann 2014) using standard bacterial annotation parameters.

### 2.6 Taxonomic Confirmation and Phylogenetic Analysis

Species‐level identification of the isolate was performed using KmerFinder v3.2 (Clausen et al. 2018; Hasman et al. 2014; Larsen et al. 2014), which compared the assembled genome against the RefSeq bacterial database using default parameters to determine the closest taxonomic match based on k‐mer composition. Average nucleotide identity (ANI) was calculated using FastANI v1.33 to quantify pairwise genomic similarity with representative *Enterobacter* genomes. Species delineation was interpreted using a ≥ 95%–96% ANI threshold, consistent with established standards in prokaryotic taxonomy (Jain et al. 2018). Tetranucleotide correlation coefficients (TETRA) were computed to confirm species affiliation further.

Phylogenetic analysis was performed using the PATRIC CodonTree pipeline (Wattam et al. 2017). Ninety‐four genomes were included in the study, and 500 conserved single‐copy protein families (PGFams) were identified and aligned. Protein sequences were aligned with MAFFT and concatenated (totaling 192,904 aligned amino acid positions across 500 proteins); corresponding nucleotide CDS alignments were concatenated (totaling 578,712 aligned nucleotides). Phylogenies were inferred with RAxML v8.2.12: protein alignments were analyzed under the JTT substitution model, and the concatenated nucleotide alignment was run with the GTRCAT model (command: raxmlHPC‐PTHREADS‐SSE3 ‐t RAxML_bipartitions. paper 2 ‐f I ‐m GTRCAT ‐n paper 2_rooted) with 100 rapid bootstrap replicates. The resulting trees were visualized using iTOL v5 (Letunic and Bork 2021). Branches with bootstrap support ≥ 70% were considered well supported.

### 2.7 Detection of Antimicrobial Resistance and Virulence Genes

AMR genes were identified using multiple databases and tools: AMRFinderPlus (Feldgarden et al. 2019), ResFinder 4.0 (Bortolaia et al. 2020), and the Comprehensive Antibiotic Resistance Database (CARD, Alcock et al. 2023)). Virulence factors were predicted using the Virulence Factor Database (VFDB;(B. Liu et al. 2022)).

### 2.8 Genomic Islands, Mobile Genetic Elements, and Plasmid Detection

Genomic islands were predicted using IslandViewer 4 (Bertelli et al. 2017) with default parameters on the web server, incorporating integrated prediction methods (IslandPath‐DIMOB, SIGI‐HMM, and IslandPick). To characterize MGEs associated with AMR loci, particularly the *bla*_CTX*‐*M‐15_, carrying contig, MGE detection was performed using MobileElementFinder (Johansson et al. 2021), applying default quality thresholds (minimum alignment coverage: 95%, minimum sequence identity: 90%, maximum truncation: 30%. The analysis focused on contig00004, which harbors *bla*_CTX*‐*M‐15_, to determine the local genetic context and identify adjacent insertion sequences, transposases, and recombination‐associated genes. Plasmid replicons were detected with PlasmidFinder 2.1 (Carattoli et al. 2014).

### 2.9 Halotolerance‐Related Gene Analysis

Genes associated with osmotic stress tolerance and halotolerance (e.g., compatible solute transporters, Na^+^/K^+^ efflux systems, mechanosensitive ion channels) were identified through KEGG pathway mapping (Kanehisa et al. 2016) and subsystem analysis in PATRIC. Comparative searches were also carried out against known halotolerance gene clusters in *Enterobacterales* using BLASTp.

## 3 Results

### 3.1 Isolation and Preliminary Identification of the Bacterial Strain

A distinct bacterial colony was recovered from mTEC agar plates inoculated with seawater collected from Laboni Beach, Cox's Bazar. The colony differed from the typical dark‐magenta *E. coli* morphology usually observed on this medium. Upon transfer to MacConkey agar, the isolate produced pink, mucoid, circular colonies due to lactose fermentation (Figure 1A). Biochemical characterization revealed an indole‐negative, citrate‐positive, and characteristic KIA reaction profile consistent with *Enterobacter* spp. (Supporting_S1 Table S1). Subsequent 16S rRNA gene sequencing confirmed the isolate as *Enterobacter* sp., showing > 99% sequence identity with reference *Enterobacter* strains in the NCBI database (Supporting_S1 Table S2).

**Figure 1:**
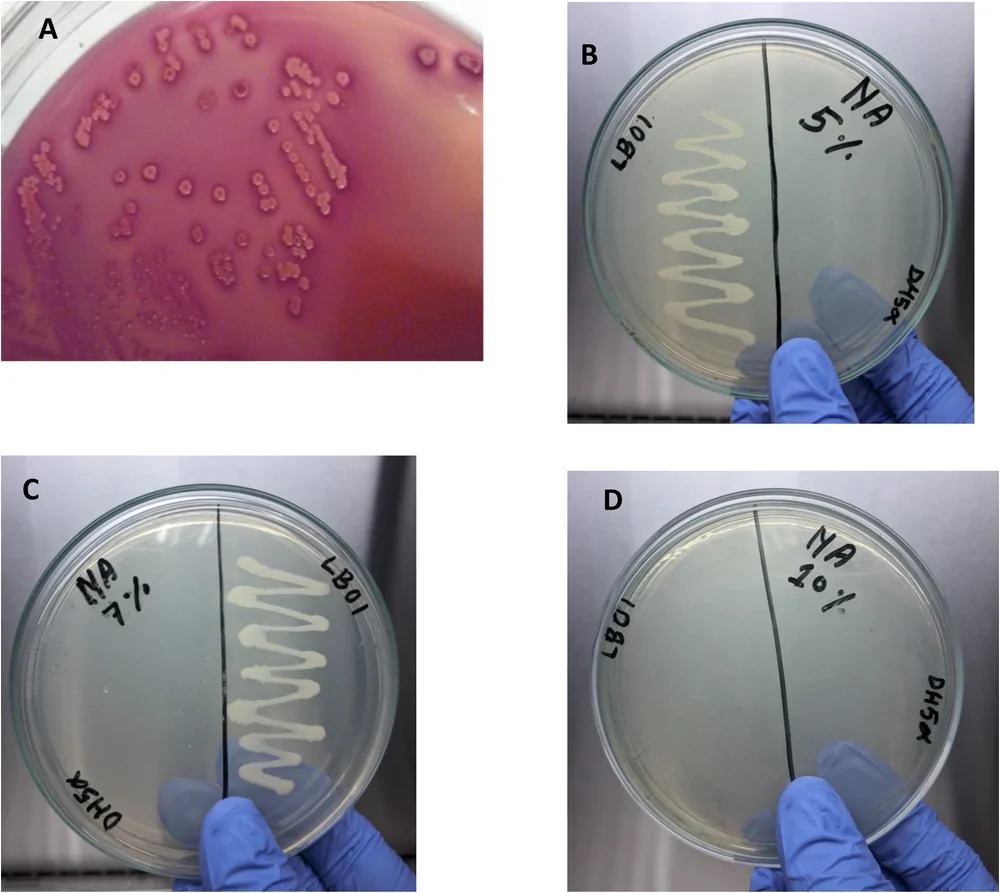
Colony morphology and salt tolerance of isolate LB01 recovered from Laboni Beach seawater. (A) Growth of isolate LB01 on MacConkey agar showing pink, mucoid, circular colonies indicative of lactose fermentation. (B–D) Salt tolerance assay on nutrient agar plates supplemented with increasing NaCl concentrations: (B) 5% NaCl, (C) 7% NaCl, and (D) 10% NaCl. The isolate showed visible growth up to 7% NaCl but failed to grow at 10% NaCl, confirming moderate halotolerance.

### 3.2 Antimicrobial Susceptibility Profile

The isolate exhibited resistance to penicillins (ampicillin and amoxicillin) and all the tested third‐generation cephalosporins (ceftazidime, ceftriaxone, cefotaxime). In contrast, it remained susceptible to fluoroquinolones (ciprofloxacin, nalidixic acid), aminoglycosides (kanamycin, gentamicin), chloramphenicol, carbapenems (imipenem), tetracycline, and trimethoprim–sulfamethoxazole (Table 1). Results were interpreted according to CLSI 2025 guidelines.

### 3.3 Salt Tolerance and Hemolytic Activity

The isolate exhibited moderate halotolerance, with growth observed in nutrient broth containing up to 7% (w/v) NaCl, while no appreciable growth occurred at 10%. Quantitative measurements showed a decline in OD_600_ values with increasing salinity (2%: 0.627; 4%: 0.476; 7%: 0.164; 10%: 0.098). These findings were consistent with plate assays, where colony formation was observed up to 7% NaCl but absent at 10% (Figure 1B–D). Control growth was consistently observed in nutrient broth containing the basal NaCl, validating the salt tolerance profile. On 5% sheep blood agar, the isolate showed γ‐hemolysis (no hemolysis), indicating the absence of detectable hemolysin production.

### 3.4 Genome Sequencing, Assembly, and Annotation

Whole‐genome sequencing of *Enterobacter* LB01 generated 21,196,074 high‐quality paired‐end Illumina reads (150 bp) with a mean Phred quality score of 39.3. After quality trimming, 21,158,862 high‐quality paired‐end reads were retained for assembly. De novo assembly produced 129 contigs with a total genome length of 4.62 Mb, a GC content of 55.05%, an N50 of 193,613 bp, and an L50 of 8 contigs, indicating a high‐quality draft genome (Table 2).

**Table 2:** Summary of genome assembly and annotation of *Enterobacter* LB01.

Feature	Value
Total genome size	4,621,806 bp
Number of contigs	129
GC content	55.05%
N50	193,613 bp
L50	8 contigs
Total CDS	4410
Hypothetical proteins	456
Proteins with functional assignment	3951
tRNAs	88
rRNAs	22
PLfam‐assigned CDS	4295
Genome completeness (CheckM)	100%
Contamination (CheckM)	0.2%

Annotation using RASTtk and Prokka identified 4,410 coding sequences (CDS), including 456 hypothetical proteins, along with 88 tRNAs and 22 rRNAs. Of the annotated CDS, 4295 were assigned to PATRIC genus‐specific families, and 3,951 proteins had functional assignments, including EC numbers, GO terms, and pathway associations. Genome quality assessment indicated 100% completeness with 0.2% contamination, confirming a robust draft genome suitable for downstream genomic analyses.

### 3.5 Taxonomic Confirmation and Phylogenetic Placement

KmerFinder analysis initially identified the isolate as *Enterobacter kobei*, showing the highest genomic similarity to reference strains available in the NCBI database. This preliminary taxonomic assignment was further corroborated using the JS species TCS approach, which indicated the closest matches with very high Z‐scores: *Enterobacter* sp. MGH 22 (0.99983), *E. kobei* ENHKU01 (0.99981), MNCRE12 (0.99981), UCI 24 (0.99977), and GN02366 (0.99973) (Table 3, Supporting_ S2, Table S3). To confirm the species‐level identification, ANI analysis was performed between the isolate and reference genomes of *E. kobei*, as well as those of *Enterobacter cloacae* ATCC strains. The ANI values with *E. kobei* reference genomes exceeded 99%. In contrast, comparisons with *E. cloacae* strains fell below the accepted species threshold (< 95%) (Figure 2), clearly distinguishing the isolate from other members of the *Enterobacter cloacae* complex (ECC). Collectively, these analyses confirm that the isolate belongs to the species *Enterobacter kobei*.

**Table 3:** Closest matches of the isolate based on JS (Jukes–Cantor) species TCS (Templeton, Crandall, and Sing) analysis (Top Five).

Closest reference strain	Taxonomic classification	Z‐Score
*Enterobacter* sp. MGH 22	*Bacteria/Pseudomonadati/Pseudomonadota/Gammaproteobacteria/Enterobacterales/Enterobacteriaceae*	0.99983
*Enterobacter kobei* ENHKU01	*Bacteria/Pseudomonadati/Pseudomonadota/Gammaproteobacteria/Enterobacterales/Enterobacteriaceae*	0.99981
*Enterobacter kobei* MNCRE12	*Bacteria/Pseudomonadati/Pseudomonadota/Gammaproteobacteria/Enterobacterales/Enterobacteriaceae*	0.99981
*Enterobacter kobei* UCI 24	*Bacteria/Pseudomonadati/Pseudomonadota/Gammaproteobacteria/Enterobacterales/Enterobacteriaceae*	0.99977
*Enterobacter kobei* GN02366	*Bacteria/Pseudomonadati/Pseudomonadota/Gammaproteobacteria/Enterobacterales/Enterobacteriaceae*	0.99973

**Figure 2:**
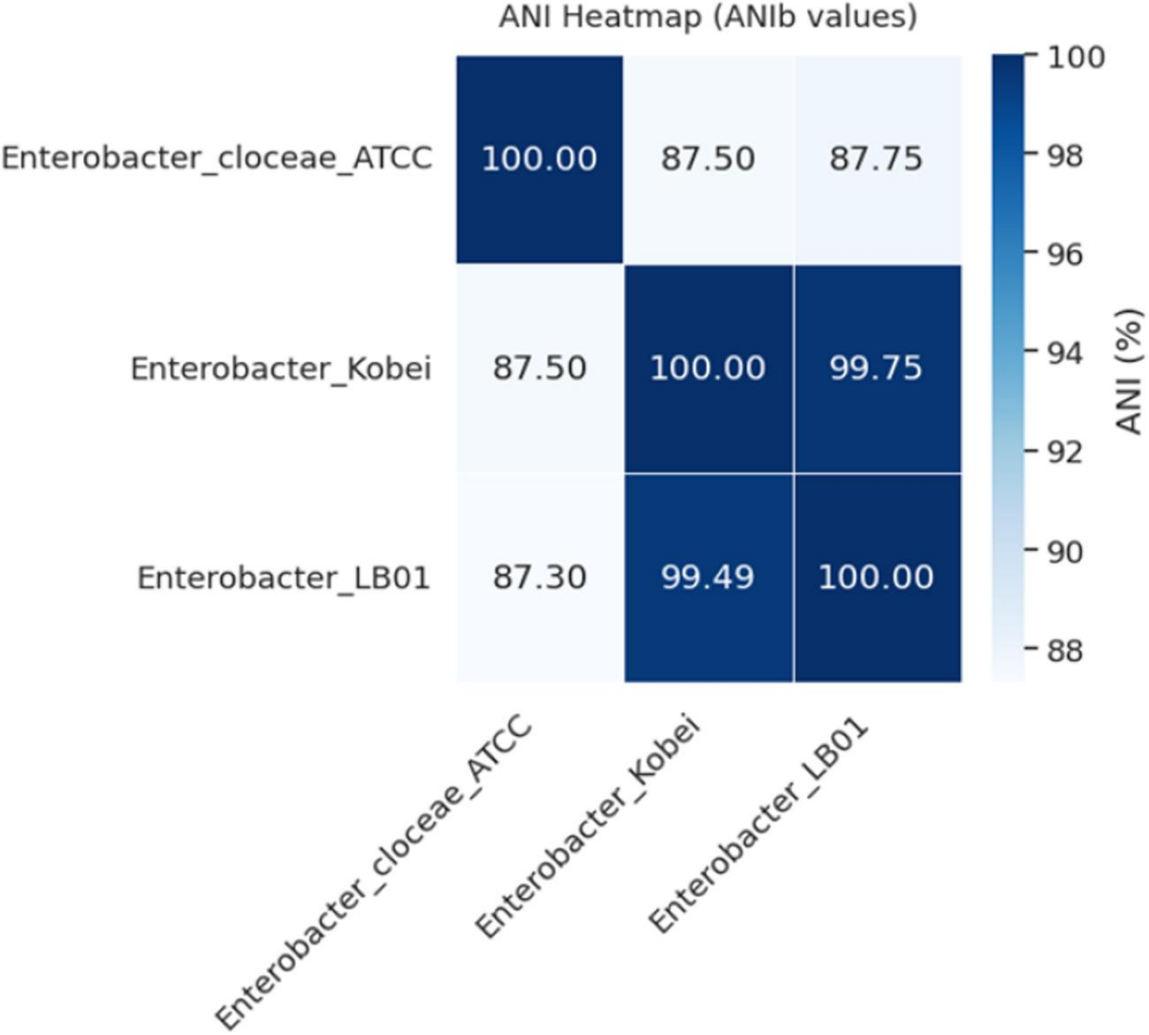
Average Nucleotide Identity (ANI) heatmap comparing *Enterobacter* sp. LB01 with closely related reference genomes. The color scale represents pairwise ANI values expressed as percentages (%), ranging from 88% to 100%. Warmer colors (e.g., dark blue) denote higher ANI values, while cooler colors (e.g., white) indicate lower similarity. ANIb analysis demonstrated that *Enterobacter* sp. LB01 shares 99.49%–99.75% identity with *Enterobacter kobei*, while showing only ~87% identity with *Enterobacter cloacae* ATCC. The inclusion of these two reference genomes highlights the close phylogenetic relationship of LB01 to *E. kobei*, supporting its taxonomic classification within this species.

Phylogenetic analysis placed LB01 within the *E. kobei* clade, clustering closely with genomes of *E. kobei* isolated from both clinical and environmental sources. The node containing LB01 and its closest relatives was supported by a bootstrap value of 100% (Figure 3), consistent with the species assignment obtained from KmerFinder, JS Species TCS, and ANI analyses.

**Figure 3:**
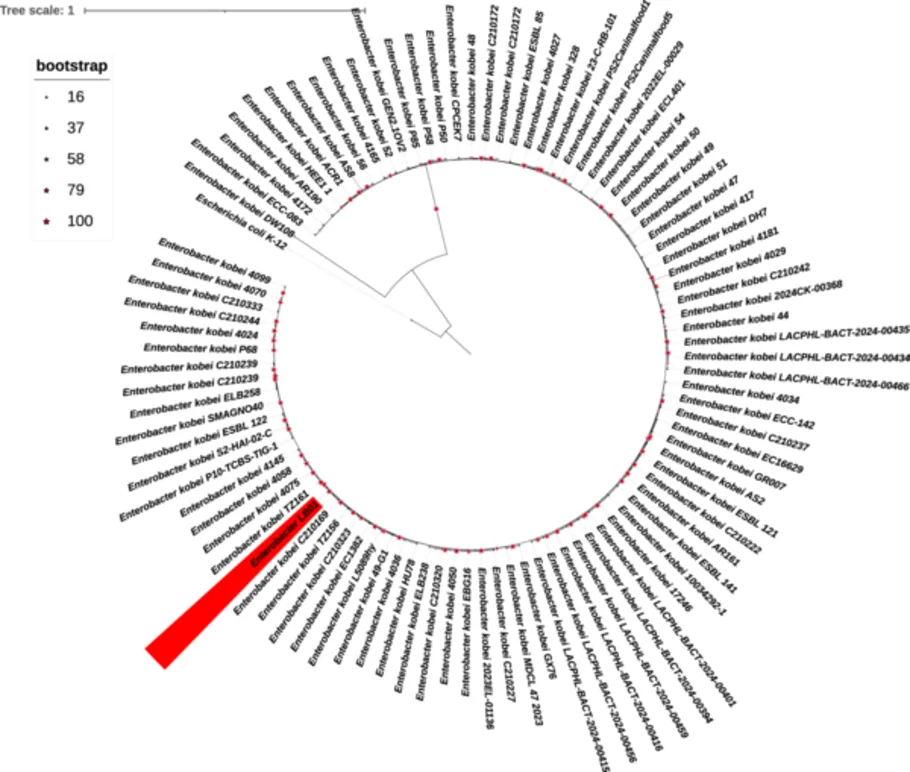
Core‐genome phylogeny of *Enterobacter* strains (94 genomes) based on 500 conserved single‐copy genes (CodonTree). Protein alignments (500 proteins; 192,904 aa) were produced and concatenated; nucleotide CDS alignments totaled 578,712 nt. Trees were reconstructed using RAxML (protein: JTT model; nucleotide: GTRCAT) with 100 bootstrap replicates. Bootstrap values ≥ 70% are shown at nodes—*Enterobacter kobei* LB01 clusters with *E. kobei* reference genomes. *E. coli* K‐12 served as the outgroup. Tree visualized in iTOL v5.

### 3.6 Antimicrobial Resistance and Virulence Determinants

Comprehensive genome analysis of *Enterobacter kobei* LB01 revealed a diverse collection of AMR and virulence‐associated genes (Figure 4). The resistome included multiple efflux systems such as *AcrAB‐TolC, AcrD, MdtBC, EmrAB*, and *OqxAB*, supported by regulatory activators like *MarA, RamA, LeuO*, and *CRP*, which work together to enhance multidrug resistance through transcriptional regulation. Additional efflux‐related proteins (*KpnE, KpnF, AdeF*) and global stress regulators (*RsmA, BaeR*) were also identified, indicating a complex regulatory network for antimicrobial tolerance.

**Figure 4:**
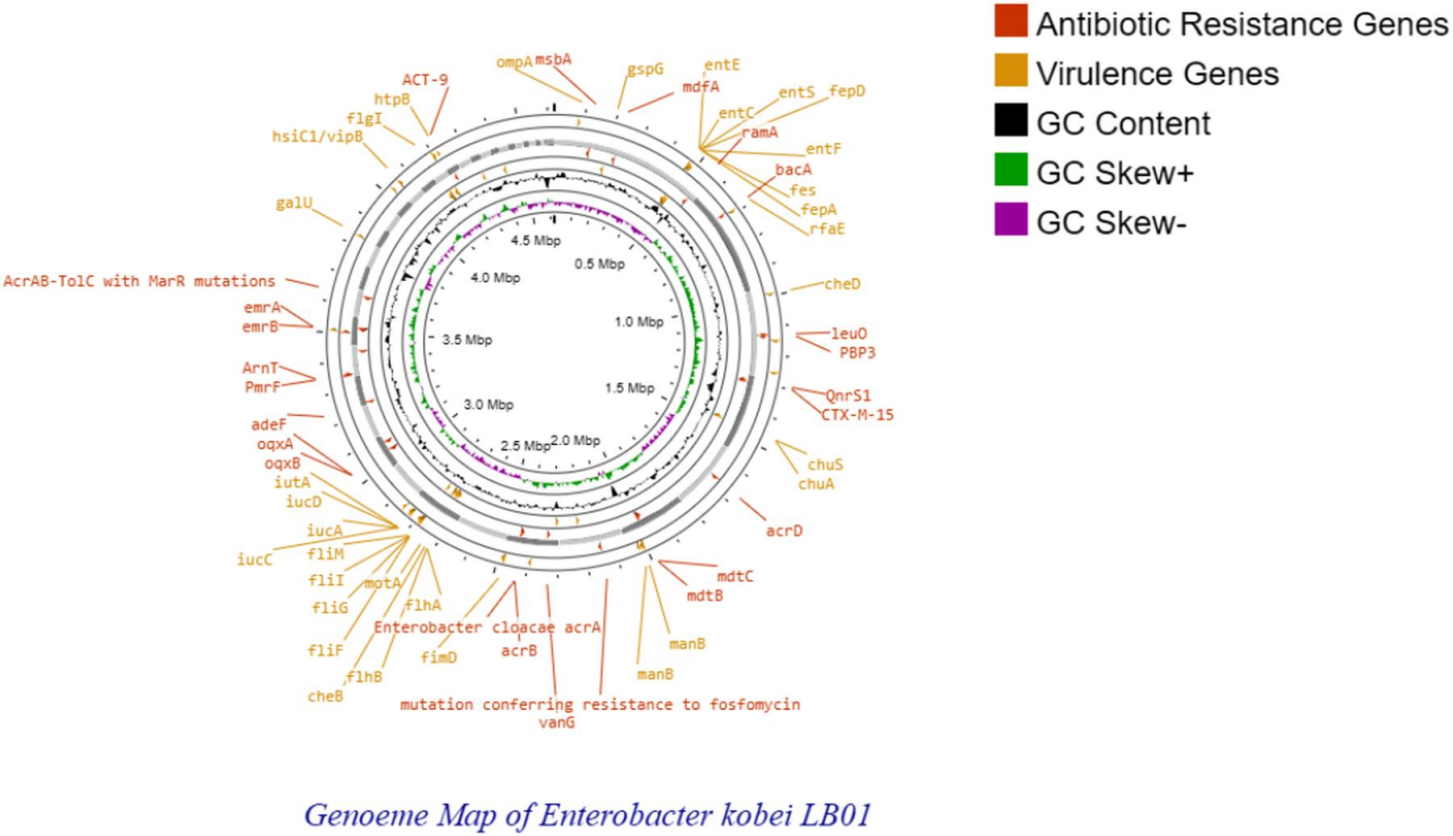
Circular genome map of *Enterobacter kobei* LB01. The genome map illustrates the distribution of antimicrobial resistance (AMR) genes, virulence factors, and GC content features. The outermost track highlights annotated genes associated with resistance (red) and virulence (brown). Inner tracks display GC content (black), GC skew+ (green), and GC skew– (purple).

β‐lactam resistance was primarily mediated by the extended‐spectrum β‐lactamase *bla*_CTX*‐*M‐15_ and *bla*_ACT‐9_, along with a PBP3 variant associated with β‐lactam resistance in *Haemophilus influenzae*. Quinolone resistance was conferred by qnrS1, while vanG encoded a glycopeptide resistance determinant. Additional genes, including *mdfA, UhpT*, and *AcrAB‐TolC* with *marR* mutations, were predicted to contribute to resistance against macrolides, fosfomycin, ciprofloxacin, and tetracycline, respectively. Collectively, these features suggest that *E. kobei* LB01 harbors a broad resistome enabling resistance to multiple antibiotic classes, including β‐lactams, fluoroquinolones, and tetracyclines.

The virulome was equally diverse, encompassing genes related to adhesion, motility, iron acquisition, secretion systems, and stress adaptation. Fimbrial operons (*fimC, fimD, fimH*) and curli fiber genes (*csgA–G*) supported surface attachment and biofilm formation, while flagellar and chemotaxis genes (*flhA, flhB, flhC, flhD, fliA–R, cheA–Z, motA*) indicated active motility and environmental responsiveness. Iron acquisition systems were extensive, including the enterobactin (*entA–F, entS*) and ferric‐enterobactin transport (*fepA–G, fes*) clusters, together with aerobactin (*iucA–D, iutA*) and heme uptake (*chuA, chuS*) operons. Outer membrane proteins such as *ompA* and *msbA* contribute to host interaction and membrane stability. Additional virulence factors, including lipopolysaccharide biosynthesis genes (*rfaD*, *rfaE*, *gmhA*, *kdsA*) and stress response elements (*clpP*, *htpB*), further support environmental persistence and potential pathogenicity.

### 3.7 Mobile Genetic Elements and Genetic Context of *bla*_CTX*‐*M‐15_

IslandViewer analysis identified 34 genomic islands across the *Enterobacter* sp. LB01 genome, all located within the chromosomal contig (Figure 5). The most prominent island (554,914–563,713 bp) encodes an integrase, restriction‐modification enzymes, and several hypothetical proteins, suggesting a prophage‐derived region associated with genome mobility and adaptation.

**Figure 5:**
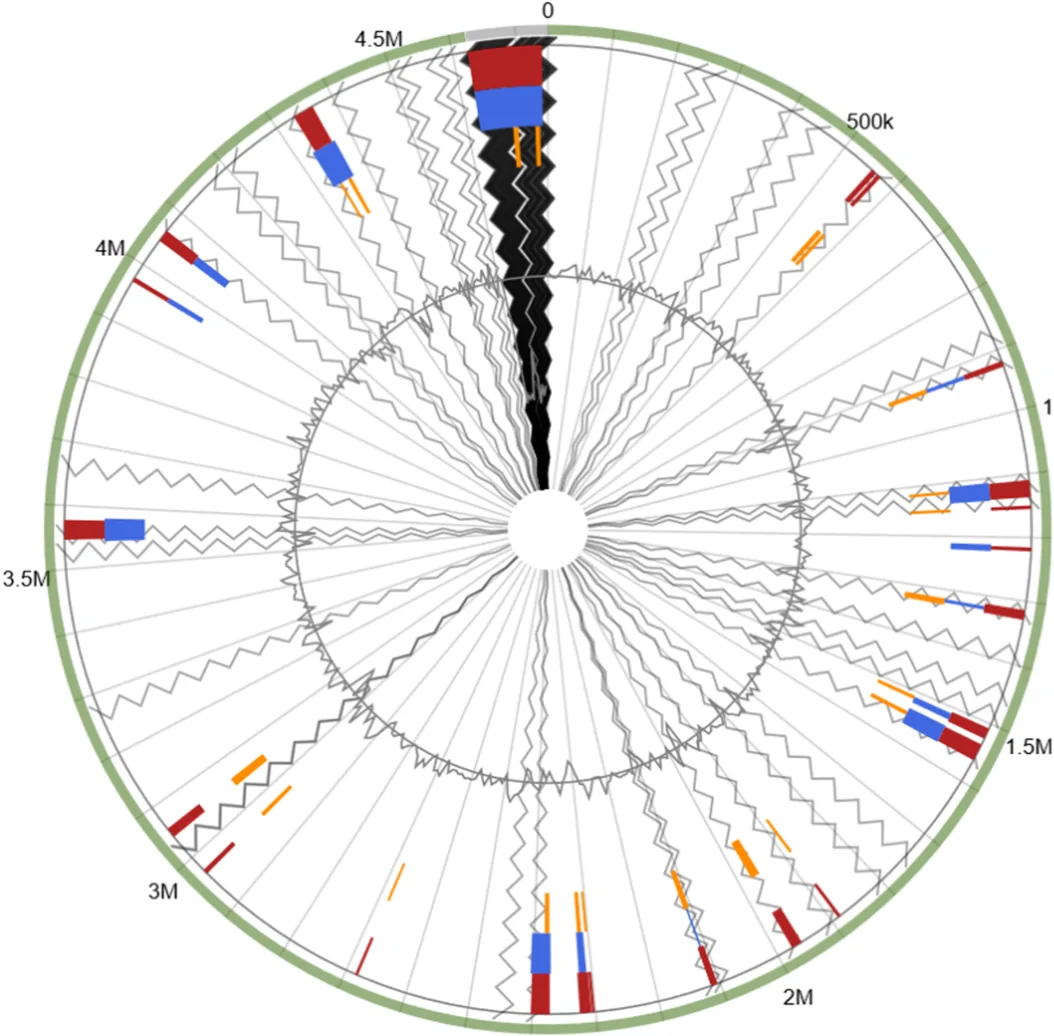
Circular representation of the *Enterobacter* sp. LB01 genome showing predicted genomic islands and associated genes. The outermost green ring represents the complete circular chromosome. Colored inner bars denote genes predicted within genomic islands: red for antibiotic resistance or mobile element–related genes, blue for phage‐associated proteins, and orange for recombination or integrase genes. The inner gray plot indicates GC skew, highlighting potential replication origin and terminus regions.

To characterize the genetic environment of the extended‐spectrum β‐lactamase gene *bla*_CTX*‐*M‐15_, MGEs were analyzed within contig00004, which harbors this resistance locus. The local gene arrangement was identified as *tnpM–tnpR–gin–qnrS1–insC1–tnpA– bla*_CTX*‐*M‐15_ *–tnpA–tnpA–tmk–holB* (Figure 6). This organization reveals that *bla*_CTX*‐*M‐15_ is embedded within a dense cluster of transposase‐ and recombinase‐related genes, suggesting a mobilizable genetic region.

**Figure 6:**
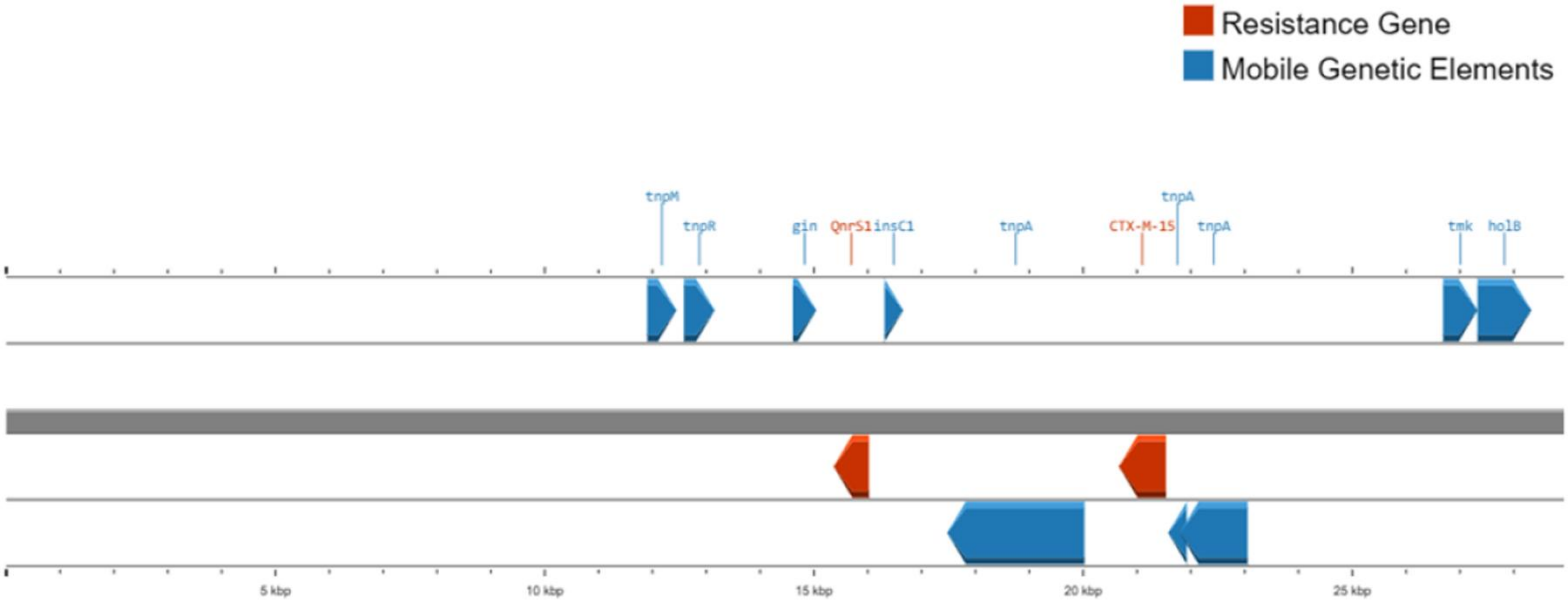
*Genetic organization of the bla*_CTX*‐*M‐15_ *region in Enterobacter kobei* LB01. The β‐lactamase gene is flanked by multiple transposases and insertion sequence elements (*tnpA*, *tnpR*, *tnpM*, *insC1*), with *qnrS1* positioned upstream. Arrows indicate gene orientation.

Upstream of *bla*_CTX*‐*M‐15_, multiple transposase genes (*tnpA*, *tnpR*, *tnpM*) and an insertion sequence element (*insC1*) were detected, representing an ISEc9/ISEcp1‐like composite transposon structure. The presence of *gin* (site‐specific recombinase) and *qnrS1* immediately upstream of this transposon further indicates that fluoroquinolone and β‐lactam resistance genes may be physically linked within the same mobile module. Downstream, housekeeping genes (*tmk*, *holB*) mark the boundary of this MGE region, defining the likely insertion limit within the chromosomal or plasmid backbone.

This arrangement—ISEc9‐associated transposases upstream and downstream of *bla*_CTX*‐*M‐15_, together with the co‐located *qnrS1*—strongly supports a mobilizable resistance cluster capable of horizontal transfer. Such ISEcp1/ISEc9*– bla*_CTX*‐*M‐15_ *–qnrS1* architectures are well documented in both clinical and environmental *Enterobacterales*, underscoring the potential for gene flow between marine and clinical reservoirs. Additionally, an IS3‐family insertion sequence (ISEhe3) was detected on a separate contig. No class 1 integrons (e.g., *intI1*) or Tn21‐family transposons were identified in the genome.

### 3.8 Functional Subsystem Annotation and Halotolerance‐Related Genomic Features

Subsystem‐based annotation of the *Enterobacter* sp. LB01 genome revealed ten major functional categories encompassing 2320 genes (Figure 7A). The majority were assigned to metabolism (886 genes, 32%), followed by protein processing (269 genes), stress response, defense, and virulence (211 genes), and energy metabolism (320 genes). Other categories included membrane transport, cellular processes, DNA and RNA processing, cell envelope biosynthesis, and regulation and signaling functions. The predominance of metabolic and stress‐associated subsystems highlights the organism's strong adaptive potential under environmental stressors, particularly saline conditions.

**Figure 7:**
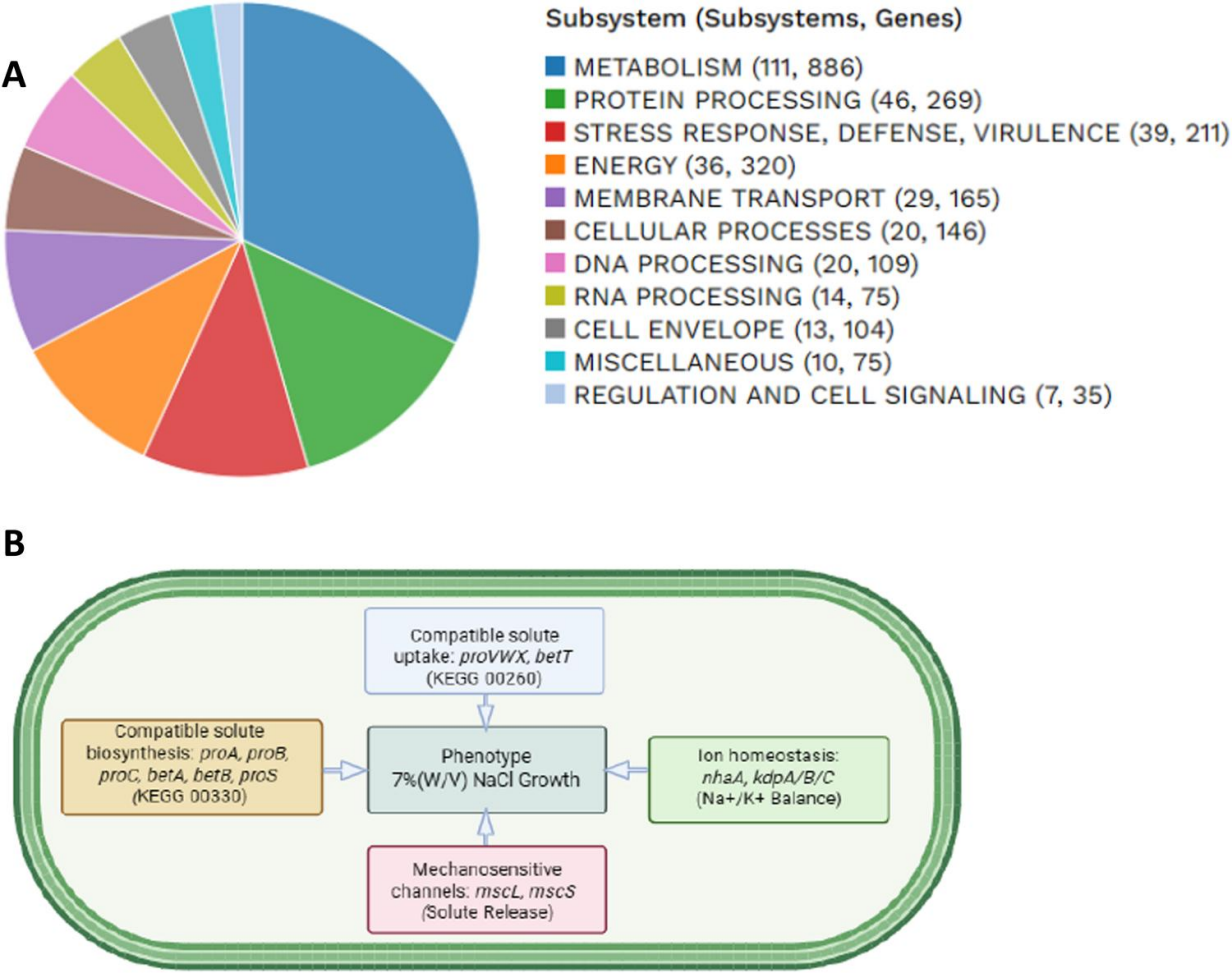
(A) Subsystem‐level functional classification of the *Enterobacter kobei* LB01 genome. Subsystem annotation revealed 10 major functional categories, dominated by metabolism (886 genes, 32%), followed by protein processing, stress response, and energy metabolism. The enrichment of metabolic and stress‐related subsystems underscores the organism's adaptive potential and halotolerance under saline environments. (B) Halotolerance network in *Enterobacter kobei* LB01 linking genomic determinants and observed phenotype. Compatible solute biosynthesis (*proA, proB, proC, betA, betB, proS*; KEGG 00330) and uptake (*proVWX, betT*; KEGG 00260) contribute to osmoprotection through accumulation of proline and glycine betaine. Ion homeostasis genes (*nhaA, kdpA/B/C*) maintain Na^+^/K^+^ balance, while mechanosensitive channels (*mscL, mscS*) enable solute release during osmotic downshock. Collectively, these mechanisms support growth up to 7% (w/v) NaCl.

The genome of *Enterobacter kobei* LB01 encodes a comprehensive repertoire of genes involved in osmotic stress adaptation and halotolerance (Table 4, Figure 7B). Multiple compatible solute transport and biosynthesis systems were identified, including the *proVWX* and *betT* transporters, which facilitate the uptake of proline and choline, respectively. Genes responsible for the biosynthesis of compatible solutes—*proA, proB*, and *proC* (for proline) and *betA* and *betB* (for glycine betaine)—were also detected, suggesting that the bacterium can both synthesize and accumulate osmoprotectants internally.

**Table 4:** Genomic determinants of halotolerance in *Enterobacter* sp. LB01 and their corresponding functional roles.

Pathway (KEGG ID)	Key genes (*Enterobacter kobei* LB01)	Function/role in halotolerance	Evidence/phenotype
Arginine & proline metabolism (00330)	*proA, proB, proC, proVWX*	Biosynthesis and uptake of proline, a major compatible solute for osmotic balance	Growth up to 7% NaCl; both biosynthetic and transport systems active
Glycine, serine & threonine metabolism (00260)	*betA, betB, betT*	Conversion of choline to glycine betaine and its uptake is a major osmoprotectant	Explains tolerance up to 7% NaCl
Alanine, aspartate & glutamate metabolism (00250)	*gdhA, gltB*	Glutamate accumulation and precursor pool maintenance for osmolyte synthesis	Supports osmolyte synthesis under salt stress
Trehalose/starch metabolism (00500)	*otsA, otsB*	Trehalose synthesis protects proteins and membranes under osmotic stress	Contributes to membrane stability
Ion transport/homeostasis	*nhaA, kdpA, kdpB, kdpC*	Na^+^/H^+^ antiporter and K^+^ uptake system for ionic balance and turgor maintenance	Maintains ion homeostasis in saline environments
Mechanosensitive channels	*mscL, mscS*	Release of solutes during osmotic downshift to prevent lysis	Enhances osmotic shock survival
Oxidative stress protection (00480)	*gshA, gshB, kat, sod*	Detoxification of reactive oxygen species generated during salt stress	Increases overall stress resilience
Central metabolism (00010, 00071)	Glycolytic and fatty acid metabolism genes	Energy production and membrane remodeling under salt stress	Supports metabolic adaptation

Ion homeostasis was mediated by the Na^+^/H^+^ antiporter (*nhaA*) and the high‐affinity K^+^ transport system (*kdpABC*), both of which are essential for maintaining ionic balance under saline stress. Additionally, the presence of mechanosensitive channel genes (*mscL* and *mscS*) indicates the ability of *Enterobacter kobei* LB01 to release intracellular solutes rapidly during sudden osmotic downshifts, thereby preventing cell lysis.

KEGG pathway mapping confirmed enrichment of arginine and proline metabolism (00330), glycine, serine, and threonine metabolism (00260), and glutathione metabolism (00480)—pathways known to enhance osmoadaptation and oxidative stress tolerance (Supporting_S3 Table S4). Collectively, these genomic features corroborate the experimentally observed phenotype, wherein *Enterobacter kobei* LB01 exhibited robust growth in media containing up to 7% NaCl but failed to grow at 10% NaCl, confirming its moderate halotolerant nature.

## 4 Discussion

The detection of a *bla*_CTX*‐*M‐15_ –harbouring *Enterobacter kobei* in a marine environment highlights the expanding ecological footprint of clinically relevant ESBL genes and underscores the role of coastal ecosystems as potential reservoirs and dissemination nodes for AMR. To our knowledge, this is the first genome‐level report of a marine *E. kobei* carrying *bla*_CTX*‐*M‐15_ from Bangladesh. This finding extends the known environmental distribution of clinically important ESBL genes into the coastal waters of South Asia and suggests that coastal aquatic ecosystems may contribute to the environmental persistence and dissemination of clinically relevant AMR (Fuentes‐Castillo et al. 2021).

The detection of *bla*_CTX*‐*M‐15_ in a marine *E. kobei* is notable for two main reasons. First, CTX‐M‐15 is among the most globally disseminated CTX‐M variants in clinical, agricultural, and wastewater settings and is strongly associated with human infections (Bevan et al. 2017); its presence in coastal bacteria indicates a spillover of clinically relevant resistance into the marine environment (Girlich et al. 2020). Second, carriage of CTX‐M enzymes by *Enterobacter* spp.—Members of the *Enterobacter cloacae* complex (ECC) have been repeatedly implicated in outbreaks and difficult‐to‐treat infections; the combination of an ECC member with CTX‐M‐15 in the marine milieu, therefore, represents a potential public‐health concern (Zhou et al. 2017).

Our genomic data showing *bla*_CTX*‐*M‐15_ adjacent to an ISEc9‐type insertion element (ISEc9/ISEc9‐like) and co‐localization with *qnrS1* strongly suggest that the ESBL determinant is in a mobilizable context (Domingues et al. 2025). Insertion sequences of the ISEcp1/ISEc9 family are well documented to mobilize adjacent *bla*_CTX*‐*M_ genes and to mediate transposition between plasmids and chromosomes, thereby facilitating horizontal gene transfer. Such genetic arrangements have been reported in clinical and environmental Enterobacteriaceae and are considered key drivers of CTX‐M dissemination (Negeri et al. 2023). Importantly, the physical linkage of *bla*_*CTX‐M‐15*_ with *qnrS1* enhances the potential for co‐selection under antibiotic pressure, as quinolone exposure can enrich for plasmids carrying *qnrS1*, indirectly maintaining the linked ESBL gene even in the absence of β‐lactam antibiotics. Consequently, quinolone use may contribute to the persistence and spread of ESBL‐mediated resistance in both aquatic and clinical environments (Poirel et al. 2005; Zhao and Huang 2026).

No plasmid replicons were identified in the assembled genome using PlasmidFinder; however, the genomic location of *bla*_*CTX‐M‐15*_ could not be conclusively determined, as replicon‐based approaches are constrained by database completeness and assembly fragmentation (Arredondo‐Alonso et al. 2017). In the absence of read coverage analysis, it remains unclear whether the gene is chromosomally integrated or located on a plasmid lacking identifiable replicon sequences.

Phenotypically, LB01 exhibited resistance to penicillins and third‐generation cephalosporins but retained susceptibility to carbapenems, aminoglycosides, tetracycline, chloramphenicol, and trimethoprim–sulfamethoxazole. This profile is concordant with the genomic content: the presence of CTX‐M‐15 and AmpC‐type *bla*ACT explains 3rd‐generation cephalosporin resistance, while the absence of carbapenemase genes matches carbapenem susceptibility. The presence of multiple efflux systems (*acrAB, acrD, mdtABC, emrAB, oqxAB*) and regulatory mutations (*MarR)* can contribute to low‐level multidrug resistance and may affect MICs for various classes under selective pressure. These genotype–phenotype relationships validate the genome assembly and highlight the clinical relevance of the isolate's resistome (Faheem et al. 2013).

The genome of LB01 encodes numerous virulence and colonization factors (fimbrial adhesins, flagellar motility genes, siderophore clusters, and heme uptake systems) that collectively support environmental persistence and potential interaction with hosts. These determinants, together with AMR genes and MGEs, mirror reports of environmental *Enterobacterales* in which virulence and resistance traits co‐occur, highlighting their potential to act as opportunistic pathogens if human exposure occurs (Anne et al. 2019). The co‐occurrence of virulence and AMR genes in coastal bacteria has been reported in several marine studies and in isolates from marine animals, reinforcing the zoonotic and ecological implications of our finding (Alves et al. 2014; Bueris et al. 2022; Di Cesare et al. 2014).

The predominance of genes associated with metabolism and stress response suggests that LB01 has a wide range of functional abilities to adapt to various environmental circumstances. LB01's ability to grow up to 7% (w/v) NaCl is supported by a clear genomic signature of osmoadaptation: compatible‐solute transporters (*proVWX, betT*), biosynthetic pathways for proline and glycine betaine (*proA/B/C, betA/B*), ion homeostasis systems (*nhaA, kdpABC*) and mechanosensitive channels (*mscL, mscS*). These systems constitute canonical halotolerance mechanisms described in diverse bacteria, enabling osmolyte accumulation and ion regulation under fluctuating salinities. These osmoadaptation factors are common among bacteria from various osmotically variable environments and are not exclusive to marine adaptation. Therefore, the halotolerance observed could result from adaptation to coastal saline conditions or from pre‐adaptation in habitats like wastewater‐impacted or estuarine environments, which also experience osmotic fluctuations. While our findings indicate that LB01 can survive in coastal salinity conditions, further genomic and ecological studies are needed to determine whether this represents true marine adaptation or pre‐adaptation from non‐marine habitats (Gunde‐Cimerman et al. 2018; Zhou et al. 2023).

Coastal ecosystems lie at the interface of terrestrial and marine systems and are frequently impacted by anthropogenic inputs (treated/untreated sewage, agricultural runoff, aquaculture effluent, and urban stormwater) (Brown et al. 2024). These inputs are well‐recognized sources of clinically important AMR genes, including CTX‐M variants and plasmid‐borne qnr genes (Goh et al. 2024). Given the global literature documenting CTX‐M‐15 and *qnrS* in environmental waters and coastal biota, wastewater contamination and fecal inputs are plausible sources for the entry of LB01 or its resistance elements into the Bay of Bengal coastal zone. The detection of mobilization elements adjacent to resistance genes supports the hypothesis that horizontal gene transfer in the coastal microbiome could further spread these determinants (Girlich et al. 2020).

The presence of a halotolerant, ESBL‐producing *E. kobei* in coastal water has multiple implications. Firstly, it indicates the environmental persistence of clinically relevant resistance genes in a habitat accessible to humans and animals through recreational contact or seafood harvest (Jinnai et al. 2024). Secondly, it suggests the potential horizontal transfer of resistance determinants to other environmental and opportunistic pathogens (Lerminiaux and Cameron 2019). Finally, it highlights the contribution of this strain to the marine resistome, which can be exchanged across ecological compartments. The co‐localization of *bla*_CTX*‐*M‐15_ with IS elements and plasmid‐associated *qnrS* increases the probability of dissemination under selective pressures such as antibiotic contamination (Poirel et al. 2005).

There are several challenges in this investigation. This study was based on a single isolate and thus couldn't quantify the prevalence or distribution of CTX‐M‐15 in the Bay of Bengal or establish the dominant sources of contamination. A short‐read assembly (129 contigs) limited full reconstruction of plasmid sequences and the precise genomic architecture (chromosomal vs plasmid location) of some AMR loci. Future work should employ long‐read sequencing platforms (e.g., Oxford Nanopore MinION or PacBio) combined with hybrid assembly to resolve plasmid architecture and the boundaries of MGEs (De Maio et al. 2019). While genomic signatures strongly indicated mobility potential, experimental demonstration of transferability (conjugation assays, plasmid curing, or transformation experiments) would be required to confirm active horizontal transfer (Sørensen et al. 2005). It would be necessary to have environmental measurements of antibiotic concentrations and source‐tracking data (e.g., wastewater markers) that would strengthen inferences about anthropogenic drivers. Finally, the absence of environmental metadata, such as antibiotic residue concentrations and wastewater source‐tracking markers, limits the interpretation of anthropogenic drivers. Incorporating targeted chemical analysis (e.g., LC–MS/MS for antibiotic quantification) (Wu et al. 2025) and microbial source tracking markers (Harwood et al. 2014) in future studies would strengthen causal inference regarding contamination sources.

Our findings added to an expanding body of evidence that coastal and marine environments are active reservoirs for clinically important AMR genes, including CTX‐M variants, and that members of the *Enterobacter cloacae* complex (including *E. kobei*) can carry and potentially disseminate such determinants. The genomic co‐occurrence of halotolerance genes, virulence determinants, efflux systems, and mobilizable resistance loci in LB01 underscored the ecological plasticity and risk posed by environmental *Enterobacterales*. Given the public health implications, coastal waters should be routinely included in AMR surveillance frameworks, particularly in regions with dense coastal populations and limited wastewater treatment infrastructure. To reduce the spread of ARGs into the environment, wastewater treatment systems should include advanced methods like UV irradiation, ozonation, or UV‐based advanced oxidation processes. These treatments need to be carefully managed, with proper UV exposure and correct oxidant levels, to effectively eliminate ARGs while avoiding unnecessary selective pressure or horizontal gene transfer (H. Liu et al. 2022; Umar 2022).

## 5 Conclusions

This study reports the first marine *Enterobacter kobei* from Bangladesh carrying the *bla*_CTX*‐*M‐15_ gene. Genomic and phenotypic analyses revealed co‐occurrence of AMR, virulence, and halotolerance traits, including a mobilizable *bla*_CTX*‐*M‐15_–qnrS1–ISEc9 cluster and osmoadaptive genes supporting growth up to 7% NaCl. These findings highlight the marine environment as a potential reservoir and dissemination route for clinically relevant resistance genes, reinforcing the need for coastal AMR surveillance within a One Health framework.

## Author Contributions

**Md Arifur Rahman:** writing – original draft; formal analysis, data curation, visualization, methodology, validation. **Rafid Muntasir Ul Azam:** formal analysis, data curation. **Md Sazib Hossain:** formal analysis, data curation. **Samina Momtaz:** funding acquisition, writing – review and editing, resources. **Anowara Begum:** resources; writing – review and editing, supervision. **Munawar Sultana:** supervision, resources, project administration, writing – review and editing, investigation, conceptualization.

## Ethics Statement

The authors have nothing to report.

## Conflicts of Interest

The authors declare no conflicts of interest.

## Policy on Using ChatGPT and Similar AI Tools

During the preparation of this manuscript, Grammarly and QuillBot were used solely for language editing, grammar correction, and improvement of sentence clarity. The authors confirm that these tools were not used for generating scientific content, data analysis, or interpretation.

## Supporting information


**Table S1:** Biochemical characterization of isolate LB01. This table summarizes the biochemical properties of the marine isolate LB01. The isolate was found to be oxidase‐negative and catalase‐positive, indicating the absence of cytochrome c oxidase and the presence of catalase enzyme activity. It did not produce indole and showed positive citrate utilization, suggesting its ability to use citrate as a sole carbon source. The isolate was negative for methyl red but positive for Voges–Proskauer, indicating acetoin production rather than mixed acid fermentation. It also demonstrated nitrate reduction capability. Kligler Iron Agar (KIA) results revealed fermentation of both glucose and lactose without gas or hydrogen sulfide (H_2_S) production.
**Table S2:** Top BLAST hits of the 16S rRNA gene sequence. This table lists the top five BLAST results obtained from the 16S rRNA gene sequence of isolate LB01. The results show high similarity (≥ 99.65% identity and 100% query coverage) with members of the genus Enterobacter, including Enterobacter kobei and Enterobacter cancerogenus. The E‐values of 0.0 indicate highly significant matches, supporting the taxonomic placement of the isolate within the Enterobacter genus.

**Table S3:** Taxonomic distribution and phylogenetic affiliation of closely related species. This table presents the taxonomic classification of closely related bacterial species identified through comparative genomic or sequence‐based analysis. It includes hierarchical classification (Domain to Family level) along with strain information and Z‐scores, indicating the relative similarity or confidence of taxonomic assignment.

**Table S4:** Functional pathway annotation of the isolate genome. This table provides an overview of metabolic and functional pathways identified in the genome of isolate LB01. Each pathway is categorized by pathway ID, name, and functional class. The table also includes annotation details such as the number of unique genomes, genes, and enzyme commission (EC) numbers associated with each pathway, along with EC and gene conservation scores, reflecting pathway completeness and conservation across related organisms.

## Data Availability

The data that support the findings of this study are openly available in the NCBI BioProject at https://www.ncbi.nlm.nih.gov/bioproject/ reference number PRJNA1354465.
