# Supplementary material for: First Report of bla CTX‐M‐15 in a Marine Thermotolerant Enterobacter Isolate From Bangladesh: Genomic Insights Into Halotolerance and Antimicrobial Resistance
Source: Microbiologyopen. 2026 Sep 29;15(5):e70425. doi: 10.1002/mbo3.70425 (PMC13624531; doi:10.1002/mbo3.70425)
Supplement: Supplementary file 1 [file MBO3-15-e70425-s002.docx]

**S1 Table 1. Biochemical characterization of isolate LB01**

| **Test** | **Result** | **Interpretation** |
| --- | --- | --- |
| **Oxidase** | − | Oxidase negative |
| **Catalase** | + | Catalase positive |
| **Indole** | − | Indole not produced |
| **Citrate utilization** | + | Citrate is utilized as the sole carbon source |
| **Methyl Red (MR)** | − | No mixed acid fermentation |
| **Voges-Proskauer (VP)** | + | Acetoin produced |
| **Nitrate reduction** | + | Nitrate is reduced to nitrite |
| **Kligler Iron Agar (KIA)** | Slant: (+) yellow; Butt: (+) yellow; Gas: −; H₂S: − | Ferments glucose and lactose, no gas or H₂S production |

**S1 Table 2. Top 5 BLAST hits of the 16S rRNA gene sequence from the isolate**

| **No.** | **Description** | **Scientific Name** | **Accession** | **Query Coverage (%)** | **% Identity** | **E-value** |
| --- | --- | --- | --- | --- | --- | --- |
| 1 | *Enterobacter sp.* AC06 gene for 16S rRNA, partial sequence | *Enterobacter sp.* | LC573961.1 | 100 | 99.65 | 0.0 |
| 2 | *Uncultured bacterium clone 5s1* 16S rRNA gene, partial sequence | *Uncultured bacterium* | DQ068844.1 | 100 | 99.65 | 0.0 |
| 3 | *Enterobacter kobei* strain BDNA-E38 16S rRNA gene, partial sequence | *Enterobacter kobei* | MN636663.1 | 100 | 99.65 | 0.0 |
| 4 | *Enterobacter cancerogenus* strain 3.2.17 chromosome, complete genome | *Enterobacter cancerogenus* | CP134403.1 | 100 | 99.65 | 0.0 |
| 5 | *Enterobacter kobei* strain 9c6 chromosome, complete genome | *Enterobacter kobei* | CP192443.1 | 100 | 99.65 | 0.0 |
