# Supplementary material for: First Report of bla CTX‐M‐15 in a Marine Thermotolerant Enterobacter Isolate From Bangladesh: Genomic Insights Into Halotolerance and Antimicrobial Resistance
Source: Microbiologyopen. 2026 Sep 29;15(5):e70425. doi: 10.1002/mbo3.70425 (PMC13624531; doi:10.1002/mbo3.70425)
Supplement: Supplementary file 2 [file MBO3-15-e70425-s003.docx]

# Table S3. Taxonomic distribution and phylogenetic affiliation of closely related species

Pos.	Species	Strain	Domain	Kingdom	Phylum	Class	Order	Family	Z-Score

1	Enterobacter sp. MGH 22	MGH 22	Bacteria	Pseudomonadati	Pseudomonadota	Gammaproteobacteria	Enterobacterales	Enterobacteriaceae	0.99983

2	Enterobacter kobei ENHKU01	ENHKU01	Bacteria	Pseudomonadati	Pseudomonadota	Gammaproteobacteria	Enterobacterales	Enterobacteriaceae	0.99981

3	Enterobacter kobei MNCRE12	MNCRE12	Bacteria	Pseudomonadati	Pseudomonadota	Gammaproteobacteria	Enterobacterales	Enterobacteriaceae	0.99981

4	Enterobacter kobei UCI 24	UCI 24	Bacteria	Pseudomonadati	Pseudomonadota	Gammaproteobacteria	Enterobacterales	Enterobacteriaceae	0.99977

5	Enterobacter kobei GN02366	GN02366	Bacteria	Pseudomonadati	Pseudomonadota	Gammaproteobacteria	Enterobacterales	Enterobacteriaceae	0.99973

6	Enterobacter kobei GN03191	GN03191	Bacteria	Pseudomonadati	Pseudomonadota	Gammaproteobacteria	Enterobacterales	Enterobacteriaceae	0.99961

7	Enterobacter kobei GN02204	GN02204	Bacteria	Pseudomonadati	Pseudomonadota	Gammaproteobacteria	Enterobacterales	Enterobacteriaceae	0.99957

8	Enterobacter kobei UCI 29	UCI 29	Bacteria	Pseudomonadati	Pseudomonadota	Gammaproteobacteria	Enterobacterales	Enterobacteriaceae	0.99957

9	Enterobacter sp. MGH 25	MGH 25	Bacteria	Pseudomonadati	Pseudomonadota	Gammaproteobacteria	Enterobacterales	Enterobacteriaceae	0.99956

10	Enterobacter kobei GN02266	GN02266	Bacteria	Pseudomonadati	Pseudomonadota	Gammaproteobacteria	Enterobacterales	Enterobacteriaceae	0.99955

11	Enterobacter kobei GN02225	GN02225	Bacteria	Pseudomonadati	Pseudomonadota	Gammaproteobacteria	Enterobacterales	Enterobacteriaceae	0.99954

12	Enterobacter kobei GN02825	GN02825	Bacteria	Pseudomonadati	Pseudomonadota	Gammaproteobacteria	Enterobacterales	Enterobacteriaceae	0.99953

13	Enterobacter kobei GN02275	GN02275	Bacteria	Pseudomonadati	Pseudomonadota	Gammaproteobacteria	Enterobacterales	Enterobacteriaceae	0.99949

14	Enterobacter kobei e1326	e1326	Bacteria	Pseudomonadati	Pseudomonadota	Gammaproteobacteria	Enterobacterales	Enterobacteriaceae	0.99949

15	Enterobacter sp. MGH 37	MGH 37	Bacteria	Pseudomonadati	Pseudomonadota	Gammaproteobacteria	Enterobacterales	Enterobacteriaceae	0.99946

16	Enterobacter kobei 8706	8706	Bacteria	Pseudomonadati	Pseudomonadota	Gammaproteobacteria	Enterobacterales	Enterobacteriaceae	0.99945

17	Enterobacter cloacae ECNIH4	ECNIH4	Bacteria	Pseudomonadati	Pseudomonadota	Gammaproteobacteria	Enterobacterales	Enterobacteriaceae	0.99944

18	Enterobacter kobei 44593	44593	Bacteria	Pseudomonadati	Pseudomonadota	Gammaproteobacteria	Enterobacterales	Enterobacteriaceae	0.99936

19	Enterobacter kobei BIDMC 67	BIDMC 67	Bacteria	Pseudomonadati	Pseudomonadota	Gammaproteobacteria	Enterobacterales	Enterobacteriaceae	0.99932

20	Enterobacter kobei GN02186	GN02186	Bacteria	Pseudomonadati	Pseudomonadota	Gammaproteobacteria	Enterobacterales	Enterobacteriaceae	0.99928

21	Enterobacter kobei DSM 13645	DSM 13645	Bacteria	Pseudomonadati	Pseudomonadota	Gammaproteobacteria	Enterobacterales	Enterobacteriaceae	0.9992

22	Enterobacter kobei 42202	42202	Bacteria	Pseudomonadati	Pseudomonadota	Gammaproteobacteria	Enterobacterales	Enterobacteriaceae	0.99913

23	Enterobacter sp. MGH 23	MGH 23	Bacteria	Pseudomonadati	Pseudomonadota	Gammaproteobacteria	Enterobacterales	Enterobacteriaceae	0.99907

24	Enterobacter kobei SMART_635	SMART_635	Bacteria	Pseudomonadati	Pseudomonadota	Gammaproteobacteria	Enterobacterales	Enterobacteriaceae	0.99895

25	Enterobacter kobei ST434:953099839	ST434:953099839	Bacteria	Pseudomonadati	Pseudomonadota	Gammaproteobacteria	Enterobacterales	Enterobacteriaceae	0.99885

26	Enterobacter kobei ST54:941713674	ST54:941713674	Bacteria	Pseudomonadati	Pseudomonadota	Gammaproteobacteria	Enterobacterales	Enterobacteriaceae	0.99864

27	Enterobacter kobei 3380STDY6027363	3380STDY6027363	Bacteria	Pseudomonadati	Pseudomonadota	Gammaproteobacteria	Enterobacterales	Enterobacteriaceae	0.99848

28	Enterobacter kobei 35730	35730	Bacteria	Pseudomonadati	Pseudomonadota	Gammaproteobacteria	Enterobacterales	Enterobacteriaceae	0.99839

29	Enterobacter chinensis 170198	170198	Bacteria	Pseudomonadati	Pseudomonadota	Gammaproteobacteria	Enterobacterales	Enterobacteriaceae	0.99696

30	Enterobacter sichuanensis WCHECL1597	WCHECL1597	Bacteria	Pseudomonadati	Pseudomonadota	Gammaproteobacteria	Enterobacterales	Enterobacteriaceae	0.99639

31	Enterobacter cloacae subsp. cloacae SMART_855	SMART_855	Bacteria	Pseudomonadati	Pseudomonadota	Gammaproteobacteria	Enterobacterales	Enterobacteriaceae	0.99624

32	Enterobacter sp. BIDMC92	BIDMC92	Bacteria	Pseudomonadati	Pseudomonadota	Gammaproteobacteria	Enterobacterales	Enterobacteriaceae	0.9962

33	Enterobacter cloacae 6kgm	6kgm	Bacteria	Pseudomonadati	Pseudomonadota	Gammaproteobacteria	Enterobacterales	Enterobacteriaceae	0.99613

34	Enterobacter cloacae GGT036	GGT036	Bacteria	Pseudomonadati	Pseudomonadota	Gammaproteobacteria	Enterobacterales	Enterobacteriaceae	0.99613

35	Enterobacter cloacae subsp. cloacae SMART_901	SMART_901	Bacteria	Pseudomonadati	Pseudomonadota	Gammaproteobacteria	Enterobacterales	Enterobacteriaceae	0.99613

36	Enterobacter sichuanensis WCHECL1597	WCHECL1597	Bacteria	Pseudomonadati	Pseudomonadota	Gammaproteobacteria	Enterobacterales	Enterobacteriaceae	0.99609

37	Enterobacter cloacae subsp. cloacae CIDEIMsCOL10	CIDEIMsCOL10	Bacteria	Pseudomonadati	Pseudomonadota	Gammaproteobacteria	Enterobacterales	Enterobacteriaceae	0.996

38	Enterobacter cloacae subsp. cloacae GN02174	GN02174	Bacteria	Pseudomonadati	Pseudomonadota	Gammaproteobacteria	Enterobacterales	Enterobacteriaceae	0.99598

39	Enterobacter cloacae subsp. cloacae GN02616	GN02616	Bacteria	Pseudomonadati	Pseudomonadota	Gammaproteobacteria	Enterobacterales	Enterobacteriaceae	0.99587

40	Enterobacter sp. 50793107	50793107	Bacteria	Pseudomonadati	Pseudomonadota	Gammaproteobacteria	Enterobacterales	Enterobacteriaceae	0.99585

41	Enterobacter cloacae subsp. cloacae SMART_1093	SMART_1093	Bacteria	Pseudomonadati	Pseudomonadota	Gammaproteobacteria	Enterobacterales	Enterobacteriaceae	0.99579

42	Enterobacter cloacae subsp. cloacae SMART_628	SMART_628	Bacteria	Pseudomonadati	Pseudomonadota	Gammaproteobacteria	Enterobacterales	Enterobacteriaceae	0.99575

43	Enterobacter cloacae DSM 30054	DSM 30054	Bacteria	Pseudomonadati	Pseudomonadota	Gammaproteobacteria	Enterobacterales	Enterobacteriaceae	0.99575

44	Enterobacter cloacae subsp. dissolvens ATCC 23373	ATCC 23373	Bacteria	Pseudomonadati	Pseudomonadota	Gammaproteobacteria	Enterobacterales	Enterobacteriaceae	0.99565

45	Enterobacter cloacae subsp. cloacae SMART_1094	SMART_1094	Bacteria	Pseudomonadati	Pseudomonadota	Gammaproteobacteria	Enterobacterales	Enterobacteriaceae	0.99562

46	Enterobacter cloacae subsp. cloacae SMART_886	SMART_886	Bacteria	Pseudomonadati	Pseudomonadota	Gammaproteobacteria	Enterobacterales	Enterobacteriaceae	0.99559

47	Enterobacter cloacae subsp. cloacae SMART_313	SMART_313	Bacteria	Pseudomonadati	Pseudomonadota	Gammaproteobacteria	Enterobacterales	Enterobacteriaceae	0.99557

48	Enterobacter cloacae subsp. dissolvens SDM	SDM	Bacteria	Pseudomonadati	Pseudomonadota	Gammaproteobacteria	Enterobacterales	Enterobacteriaceae	0.99557

49	Enterobacter cloacae subsp. dissolvens GN02534	GN02534	Bacteria	Pseudomonadati	Pseudomonadota	Gammaproteobacteria	Enterobacterales	Enterobacteriaceae	0.99555

50	Enterobacter sichuanensis 35699	35699	Bacteria	Pseudomonadati	Pseudomonadota	Gammaproteobacteria	Enterobacterales	Enterobacteriaceae	0.99542

51	Enterobacter cloacae B2	B2	Bacteria	Pseudomonadati	Pseudomonadota	Gammaproteobacteria	Enterobacterales	Enterobacteriaceae	0.99538

52	Enterobacter cloacae ATCC 13047	ATCC 13047	Bacteria	Pseudomonadati	Pseudomonadota	Gammaproteobacteria	Enterobacterales	Enterobacteriaceae	0.99533

53	Enterobacter cloacae ATCC 13047	ATCC 13047	Bacteria	Pseudomonadati	Pseudomonadota	Gammaproteobacteria	Enterobacterales	Enterobacteriaceae	0.9953

54	Enterobacter cloacae subsp. cloacae CIP60.85T	CIP60.85T	Bacteria	Pseudomonadati	Pseudomonadota	Gammaproteobacteria	Enterobacterales	Enterobacteriaceae	0.99525

55	Enterobacter cloacae subsp. cloacae 42324	42324	Bacteria	Pseudomonadati	Pseudomonadota	Gammaproteobacteria	Enterobacterales	Enterobacteriaceae	0.99517

56	Enterobacter cloacae ATCC 13047	ATCC 13047	Bacteria	Pseudomonadati	Pseudomonadota	Gammaproteobacteria	Enterobacterales	Enterobacteriaceae	0.99499

57	Enterobacter cloacae subsp. cloacae ATCC 13047	ATCC 13047	Bacteria	Pseudomonadati	Pseudomonadota	Gammaproteobacteria	Enterobacterales	Enterobacteriaceae	0.99483

58	Enterobacter sp. BIDMC 26	BIDMC 26	Bacteria	Pseudomonadati	Pseudomonadota	Gammaproteobacteria	Enterobacterales	Enterobacteriaceae	0.99482

59	Enterobacter cloacae subsp. cloacae ATCC 13047	ATCC 13047	Bacteria	Pseudomonadati	Pseudomonadota	Gammaproteobacteria	Enterobacterales	Enterobacteriaceae	0.99477

60	Enterobacter cloacae DSM 30054	DSM 30054	Bacteria	Pseudomonadati	Pseudomonadota	Gammaproteobacteria	Enterobacterales	Enterobacteriaceae	0.99476

61	Enterobacter cloacae FDAARGOS 1431	FDAARGOS 1431	Bacteria	Pseudomonadati	Pseudomonadota	Gammaproteobacteria	Enterobacterales	Enterobacteriaceae	0.99476

62	Enterobacter cloacae subsp. cloacae ST436:951358951	ST436:951358951	Bacteria	Pseudomonadati	Pseudomonadota	Gammaproteobacteria	Enterobacterales	Enterobacteriaceae	0.99462

63	Enterobacter hormaechei subsp. hoffmannii SMART_1105	SMART_1105	Bacteria	Pseudomonadati	Pseudomonadota	Gammaproteobacteria	Enterobacterales	Enterobacteriaceae	0.99452

64	Enterobacter mori LMG 25706	LMG 25706	Bacteria	Pseudomonadati	Pseudomonadota	Gammaproteobacteria	Enterobacterales	Enterobacteriaceae	0.99447

65	Enterobacter ludwigii GN04920	GN04920	Bacteria	Pseudomonadati	Pseudomonadota	Gammaproteobacteria	Enterobacterales	Enterobacteriaceae	0.99446

66	Enterobacter hormaechei subsp. hoffmannii DSM 14563	DSM 14563	Bacteria	Pseudomonadati	Pseudomonadota	Gammaproteobacteria	Enterobacterales	Enterobacteriaceae	0.99438

67	Enterobacter hormaechei subsp. hoffmannii SMART_266	SMART_266	Bacteria	Pseudomonadati	Pseudomonadota	Gammaproteobacteria	Enterobacterales	Enterobacteriaceae	0.99436

68	Enterobacter hormaechei MNCRE55	MNCRE55	Bacteria	Pseudomonadati	Pseudomonadota	Gammaproteobacteria	Enterobacterales	Enterobacteriaceae	0.99427

69	Enterobacter hormaechei subsp. hoffmannii 35025	35025	Bacteria	Pseudomonadati	Pseudomonadota	Gammaproteobacteria	Enterobacterales	Enterobacteriaceae	0.99424

70	Enterobacter sp. MGH 9	MGH 9	Bacteria	Pseudomonadati	Pseudomonadota	Gammaproteobacteria	Enterobacterales	Enterobacteriaceae	0.99423

71	Enterobacter hormaechei subsp. hoffmannii 35030	35030	Bacteria	Pseudomonadati	Pseudomonadota	Gammaproteobacteria	Enterobacterales	Enterobacteriaceae	0.99423

72	Enterobacter hormaechei subsp. hoffmannii 35014	35014	Bacteria	Pseudomonadati	Pseudomonadota	Gammaproteobacteria	Enterobacterales	Enterobacteriaceae	0.99423

73	Enterobacter sp. BWH 39	BWH 39	Bacteria	Pseudomonadati	Pseudomonadota	Gammaproteobacteria	Enterobacterales	Enterobacteriaceae	0.99416

74	Enterobacter sp. MGH127	MGH127	Bacteria	Pseudomonadati	Pseudomonadota	Gammaproteobacteria	Enterobacterales	Enterobacteriaceae	0.99416

75	Enterobacter sp. MGH 12	MGH 12	Bacteria	Pseudomonadati	Pseudomonadota	Gammaproteobacteria	Enterobacterales	Enterobacteriaceae	0.99411

76	Enterobacter sp. MGH 5	MGH 5	Bacteria	Pseudomonadati	Pseudomonadota	Gammaproteobacteria	Enterobacterales	Enterobacteriaceae	0.99411

77	Enterobacter hormaechei subsp. hoffmannii GN02358	GN02358	Bacteria	Pseudomonadati	Pseudomonadota	Gammaproteobacteria	Enterobacterales	Enterobacteriaceae	0.99409

78	Enterobacter cloacae ECNIH5	ECNIH5	Bacteria	Pseudomonadati	Pseudomonadota	Gammaproteobacteria	Enterobacterales	Enterobacteriaceae	0.99409

79	Enterobacter sp. MGH85	MGH85	Bacteria	Pseudomonadati	Pseudomonadota	Gammaproteobacteria	Enterobacterales	Enterobacteriaceae	0.99407

80	Enterobacter sp. MGH 11	MGH 11	Bacteria	Pseudomonadati	Pseudomonadota	Gammaproteobacteria	Enterobacterales	Enterobacteriaceae	0.99404

81	Enterobacter hormaechei subsp. hoffmannii 35032	35032	Bacteria	Pseudomonadati	Pseudomonadota	Gammaproteobacteria	Enterobacterales	Enterobacteriaceae	0.99401

82	Enterobacter hormaechei subsp. hoffmannii 35683	35683	Bacteria	Pseudomonadati	Pseudomonadota	Gammaproteobacteria	Enterobacterales	Enterobacteriaceae	0.99401

83	Enterobacter sp. MGH 8	MGH 8	Bacteria	Pseudomonadati	Pseudomonadota	Gammaproteobacteria	Enterobacterales	Enterobacteriaceae	0.99399

84	Enterobacter hormaechei subsp. hoffmannii CHS 78	CHS 78	Bacteria	Pseudomonadati	Pseudomonadota	Gammaproteobacteria	Enterobacterales	Enterobacteriaceae	0.99397

85	Enterobacter sp. MGH 26	MGH 26	Bacteria	Pseudomonadati	Pseudomonadota	Gammaproteobacteria	Enterobacterales	Enterobacteriaceae	0.99395

86	Enterobacter hormaechei subsp. hoffmannii ECR091	ECR091	Bacteria	Pseudomonadati	Pseudomonadota	Gammaproteobacteria	Enterobacterales	Enterobacteriaceae	0.99394

87	Enterobacter ludwigii EcWSU1	EcWSU1	Bacteria	Pseudomonadati	Pseudomonadota	Gammaproteobacteria	Enterobacterales	Enterobacteriaceae	0.99391

88	Enterobacter hormaechei subsp. hoffmannii GN02768	GN02768	Bacteria	Pseudomonadati	Pseudomonadota	Gammaproteobacteria	Enterobacterales	Enterobacteriaceae	0.9939

89	Enterobacter hormaechei subsp. hoffmannii SMART_309	SMART_309	Bacteria	Pseudomonadati	Pseudomonadota	Gammaproteobacteria	Enterobacterales	Enterobacteriaceae	0.9939

90	Enterobacter hormaechei subsp. hoffmannii 36796	36796	Bacteria	Pseudomonadati	Pseudomonadota	Gammaproteobacteria	Enterobacterales	Enterobacteriaceae	0.9939

91	Enterobacter hormaechei subsp. hoffmannii UCI 50	UCI 50	Bacteria	Pseudomonadati	Pseudomonadota	Gammaproteobacteria	Enterobacterales	Enterobacteriaceae	0.9938

92	Enterobacter sp. BWH 27	BWH 27	Bacteria	Pseudomonadati	Pseudomonadota	Gammaproteobacteria	Enterobacterales	Enterobacteriaceae	0.99374

93	Enterobacter ludwigii UW5	UW5	Bacteria	Pseudomonadati	Pseudomonadota	Gammaproteobacteria	Enterobacterales	Enterobacteriaceae	0.99372

94	Enterobacter sp. MGH 4	MGH 4	Bacteria	Pseudomonadati	Pseudomonadota	Gammaproteobacteria	Enterobacterales	Enterobacteriaceae	0.9937

95	Enterobacter hormaechei e742	e742	Bacteria	Pseudomonadati	Pseudomonadota	Gammaproteobacteria	Enterobacterales	Enterobacteriaceae	0.9937

96	Enterobacter ludwigii Hanford	Hanford	Bacteria	Pseudomonadati	Pseudomonadota	Gammaproteobacteria	Enterobacterales	Enterobacteriaceae	0.99368

97	Enterobacter intestinihominis CLA-AC-H004	CLA-AC-H004	Bacteria	Pseudomonadati	Pseudomonadota	Gammaproteobacteria	Enterobacterales	Enterobacteriaceae	0.99366

98	Enterobacter hormaechei subsp. hoffmannii UCICRE 3	UCICRE 3	Bacteria	Pseudomonadati	Pseudomonadota	Gammaproteobacteria	Enterobacterales	Enterobacteriaceae	0.99363

99	Enterobacter hormaechei subsp. hoffmannii ECNIH3	ECNIH3	Bacteria	Pseudomonadati	Pseudomonadota	Gammaproteobacteria	Enterobacterales	Enterobacteriaceae	0.99359

100	Enterobacter ludwigii GN02226	GN02226	Bacteria	Pseudomonadati	Pseudomonadota	Gammaproteobacteria	Enterobacterales	Enterobacteriaceae	0.99356
